# TOM2024: Datasets of tomato, onion, and maize images for developing pests and diseases AI-based classification models

**DOI:** 10.1016/j.dib.2025.111357

**Published:** 2025-02-06

**Authors:** Obed Appiah, Kwame Oppong Hackman, Son Diakalia, Audrey Kantz Dossou Codjia, Momo Bêbê, Valentin Ouedraogo, Belko Abdoul Aziz Diallo, Kisito Gandji, Damoue Abdoul-Karim, Kehinde Olufunso Ogunjobi, Gaston Dabire, Charles Lamoussa Sanou, David Anaafo, Emmanuel Ramde

**Affiliations:** a Competence Centre, WASCAL, Ouagadougou, Burkina Faso; b Gaoua University Center (CUG), Directorate of Plant Protection and Packaging (DPVC/MARAH), Burkina Faso; c Afrique Géosciences, Ouagadougou, Burkina Faso; d Nazi BONI University, Gaoua University Center, Bobo-Dioulasso, Burkina Faso; e Department of Computer Science and Informatics, UENR, Sunyani, Ghana; f Department of Geography and Sustainability, UENR, Sunyani, Ghana

**Keywords:** Dataset, Maize dataset, Onion dataset, Tomato dataset, Deep learning, Classification, Pests and disease detection

## Abstract

The advancement of digital technologies has significantly impacted plant pest and disease management, yet gaps remain, especially in developing regions. This paper introduces the TOM2024 dataset, a comprehensive collection of high-resolution images designed to enhance pest and disease identification of maize, tomato, and onion crops. The dataset encompasses 25,844 raw images and over 12,000 labeled images, categorized into 30 classes (healthy crop, infested crop, and pest) across the three cropping systems. Acquired through meticulous fieldwork in Burkina Faso using high-resolution cameras, the dataset includes diverse environmental conditions and crop stages, ensuring a robust resource for AI model training and validation. The dataset is segmented into three categories: processed images (Category A), selected images with augmentation (Category B), and an online repository with over 25,000 raw images (Category C). Category A and B features images of crops affected by 21 distinct pests and diseases. This dataset addresses critical gaps in existing collections by offering extensive coverage and high-resolution imagery that can be used to developed AI models for automatic identification and classification of pests and diseases that affects crops. TOM2024’s versatility extends to research, educational purposes, and the practical application of digital tools in agriculture thereby contributes to the advancement of precision agriculture, sustainable agricultural practices, and food security globally.

Specifications Table

**Table:** 

Subject	Artificial Intelligence (AI) / Machine Learning (ML) / Deep Learning (DL) / Convolutional Neural Network (CNN).
Specific subject area	Identification and Classification of Crop Pests and Diseases.
Type of data	Images of Plants Affected by Pests and Diseases.
Data collection	The dataset was compiled by capturing high-resolution images of tomato, onion, and maize crops, focusing on both healthy specimens and those affected by various pests and diseases. The images were taken using a high-resolution camera, producing diverse dimensions, including but not limited to 4128 × 3096 pixels. For tomato crops, the dataset includes images categorized into 14 distinct classes: 2 for pests (Helicoverpa armigera, Tuta absoluta) and 12 for diseases and healthy instances (Virosis, Healthy leaf, Healthy fruit, Mite, Alternaria, Bacterial wilt, Blossom end rot, Late blight, Sunburn, Excess nitrogen, Fusarium, Alternaria Mite). For onion crops, there are 6 classes: 1 for pests (Caterpillars) and 5 for diseases and healthy instances (Fusarium, Healthy leaf, Alternaria, Virosis, Bulb rot). For maize crops, the dataset contains 10 classes: 3 for pests (Fall Armyworm Activity, Armyworm, Aphids) and 7 for diseases and healthy leaf (Stripe, Healthy leaf, Curvularia, Helminthosporiosis, Virose, Rust, Abiotic disease). The images were captured under various lighting conditions and different backgrounds, ranging from white and dark to illuminated and natural settings.
Data source location	West African Science Service Centre on Climate Change and Adapted Land Use (WASCAL) 6 BP 9507 Ouagadougou, Burkina Faso Tel: +226 25375423 Email: secretariat_cc@wascal.org Website: www.wascal.org.
Data accessibility	Repository name: TOM2024 Data identification number: doi: 10.17632/3d4yg89rtr.1 Direct URL to data: https://data.mendeley.com/datasets/3d4yg89rtr/1
Related research article	https://www.mdpi.com/2077-0472/14/8/1252.

## 1 Value of the Data

• This extensive dataset includes 25,844 raw images and 12,227 labeled images (created through cropping of raw images) of tomato, onion, and maize crops. It serves as a vital resource for identifying and managing crop pests and diseases, supporting efforts to protect farmers' yields and strengthen food security.
• With images classified into 30 distinct categories, the dataset provides detailed visual insights into crop health and conditions, which significantly enhances the accuracy and reliability of pest and disease identification.
• By promoting early and accurate pest and disease detection, this dataset supports sustainable agricultural practices, helping to minimize excessive pesticide use and thereby contributing to environmental and economic sustainability.
• The accessibility of this freely downloadable dataset creates valuable opportunities for researchers and organizations to develop innovative digital tools for effective pest and disease management, directly benefiting farmers.
• It is particularly valuable for training, testing, and validating machine learning models focused on plant pest and disease classification, making a substantial contribution to precision agriculture.
• The project's website offers a powerful search feature, allowing users to filter raw data by region, commune, crop type, and other criteria. This unique versatility not only aids model development but also enhances its utility for educational and extension services, setting it apart from existing datasets.

## 2 Background

Digital technologies such as Artificial Intelligent (AI) have revolutionized the field of plant pest and disease identification [[1], [2], [3], [4]], especially, the use of mobile apps for quick and accurate diagnoses of diseases, critical for preventing crop losses [5]. However, vulnerable countries such as Burkina Faso and other African countries, where crop losses can sometimes reach up to fifty percent (50 %), due to insects, pathogens, nematodes, and weeds, significantly threatens food security across the continent [6,7]. These countries have not been able to fully adopt AI tools to accurately identify and classify issues in crop, largely due to the fact that developing AI models require vast and diverse datasets [[8], [9], [10], [11]]. Acquiring such datasets can be challenging, particularly due to the variability in crop types, environmental conditions, and agricultural practices across different regions of the world affecting performance [[12], [13], [14]]. As such most datasets are created in a controlled lighting environment, limiting its usefulness in broader agricultural applications [16]. To overcome these limitations, creating a dataset that encompasses a broader range of lightening and background conditions is essential to augment the existing datasets to help improve the performance of the AI models, especially for usage on farms.

The TOM2024 dataset encompass high-resolution images and cover a variety of conditions to improve the robustness and accuracy of deep learning models. By incorporating detailed categories and diverse environmental conditions, this dataset will provide a more comprehensive resource for researchers and practitioners. The dataset comprises 12,227images across 30 distinct classes grouped into 3 main cropping systems (Tomato, Onion and Maize). TOM2024 is organized into three (3) main categories. CATEGORY A consists of 12,227 labelled images, CATEGORY B contains 40,762 images obtained by applying image augmentation techniques on some of the images and CATEGORY C is an interactive repository containing over 25,000 unprocessed images. The dataset can be used in developing effective DL models or as an addition to existing dataset of similar characteristics to develop effective models, leading to enhanced performance, ultimately contributing to improved crop management and food security.

## 3 Data Description

TOM2024 [17] consists of a comprehensive collection of high-resolution images meticulously categorized by crop type, pest, and disease. This dataset is segmented into three distinct categories, providing a total of 25,844 raw images and 12,227 preprocessed images. Each image has been processed to maintain high quality and uniformity, ensuring suitability for advanced image processing tasks. The preprocessed images have fixed aspect ratio to ensure uniformity during the training of Convolutional Neural Networks (CNNs). The images in this dataset were captured under diverse environmental conditions across three(3) regions in Burkina Faso, ensuring variability in lighting, angles, and backgrounds. This diversity enhances the robustness of the dataset, making it suitable for training and evaluating AI models in real-world agricultural scenarios. Each category folder is organized into specific subfolders:

• **Category A**: This subfolder contains JPG images of tomato, onion, and maize crops. The images feature various conditions, from natural outdoor settings with varying environmental factors to controlled environments with consistent lighting. This variety includes different pests and diseases, providing a comprehensive training ground for machine learning models.
• **Category B**: This subfolder contains augmented images derived from a subset of Category A. These images were generated using several augmentation techniques, such as rotation, flipping, brightness adjustment, and more, to simulate real-world variability. This category ensures that models trained on this data are robust and can generalize well to different conditions.
• **Category C**: This is an online repository allowing users to search through the extensive collection of raw images by regions, communes, and other criteria. The repository offers easy access and retrieval of images, which can be downloaded when needed. The images in this category are organized to facilitate research and educational applications.

### 3.1 Technical details

• **Image Resolution and Quality**: Each image in the dataset is of high resolution, capturing the fine details necessary for precise pest and disease identification. The high resolution supports detailed feature extraction and model training, critical for developing AI models. Images in the dataset are of varying sizes, but with 1:1 aspect ratio.
• **Lighting and Angle Variations**: The dataset includes images taken under various lighting (sunlight) conditions, from bright sunlight to shaded areas, and from multiple angles to ensure that models trained on this data can handle real-world variability.
• **Background Complexity**: The dataset includes images with varying degrees of background complexity, from simple, uncluttered backgrounds to more complex, textured ones. This variability helps develop models that can generalize well to different real-world conditions.
• **Class Diversity**: The dataset comprises various categories covering multiple pest and disease types across three major crops: tomato, onion, and maize. This diversity enhances the dataset's applicability across various agricultural tasks. Details of the various classes are presented on Table 1.

**Table 1:** Category A.

Crop	Image categories	Number of classes	Name of pest / disease	Number of raw images	Number of processed images	Types of image processing
Tomato	Pest	2	Helicoverpa Harmigera	278	278	Cropping
			Tuta Absoluta	19	19	Cropping
	Diseases	12	Virosis	1542	1542	Cropping
			Healthy_Leaf	782	782	Cropping
			Healthy Fruit	552	552	Cropping
			Mite	390	390	Cropping
			Alternaria	322	322	Cropping
			Bacterial_floundering	252	252	Cropping
			Blossom_end_rot	118	118	Cropping
			Tomato_late_blight	74	74	Cropping
			Sunburn	51	51	Cropping
			Nitrogen_Excess	43	43	Cropping
			Fusarium	18	18	Cropping
			Alternaria_ Mite	8	8	Cropping
Onion	Pest	1	Caterpillars	879	879	Cropping
	Diseases	5	Fusarium	738	738	Cropping
			Healthy_Leaf	679	679	Cropping
			Alternaria	515	515	Cropping
			Virosis	203	203	Cropping
			Bulb_blight	30	30	Cropping
Maize	Pest	3	Spodoptera frugiperda (Fall Army Worm)_Activity	616	616	Cropping
			Spodoptera frugiperda	600	600	Cropping
			Aphids	4	4	Cropping
	Diseases	7	Stripe	2190	2190	Cropping
			Healthy_Leaf	585	585	Cropping
			Curvularia	256	256	Cropping
			Helminthosporiosis	159	159	Cropping
			Virosis	148	148	Cropping
			Rust	99	99	Cropping
			Abiotic_Diseases	77	77	Cropping

### 3.2 Data structure

**Fig. 1:**
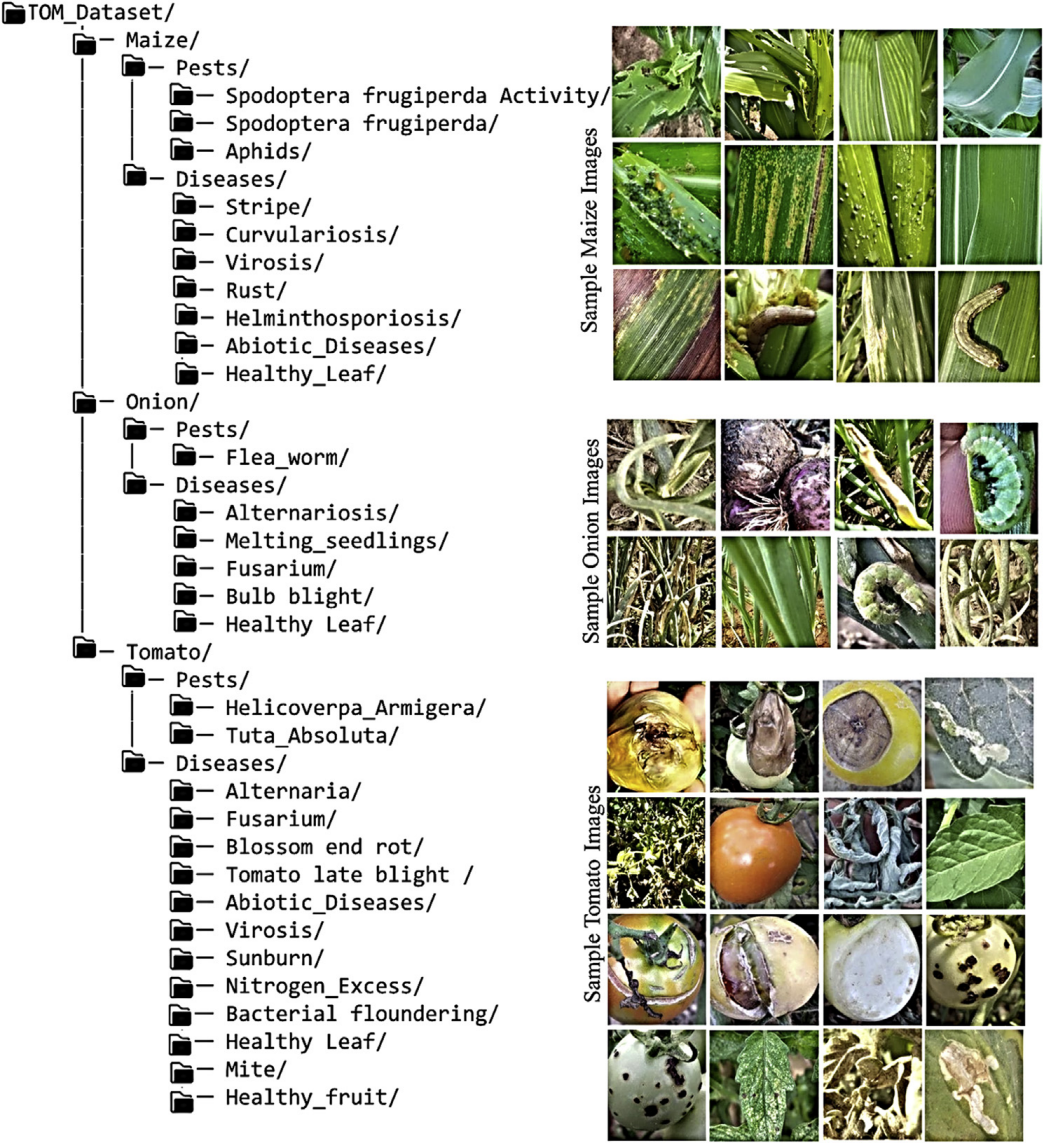
Category A with sample processed images.
• *File Naming Convention*: We adopted a non-convention naming systems for the files in the dataset. The original file name which is made up of 13 digits and 9 digits randomly generated number were concatenated to get the name for each processed file. This ensures that, no two or more files have the same names irrespective of the class they belong. This technique was adopted to enable traceability of processed images. For example, image name 158809554_1660552830471.jpg, suggests that the processed image was extracted from image with name 1660552830471.jpg from the raw images.

• *Organized Directory Structure*: The dataset is organized into 3 categories (Category A, B, and C). Category A and B are downloadable zip files or folders, while Category C is an online repository that allows the user to search through over 25,000 raw images using regions, communes, etc., after which the retrieved images can be downloaded. The datasets are organized into a well-defined hierarchical structure, facilitating ease of access and usability for various applications, including AI model development, research, and educational purposes. Fig. 1 presents the structure of Category A of the datasets and sample images

#### 3.2.1 TOM2024: category A

Table 1 presents the various pests and diseases, and the number of images processed. The table consist of the various classes (pests, diseases, and healthy instances) and the number of processed data. The type of image processing techniques used to extract the image is also indicated. The folder structure of CategoryA with sample images is illustrated in Fig. 1.

**Fig. 2:**
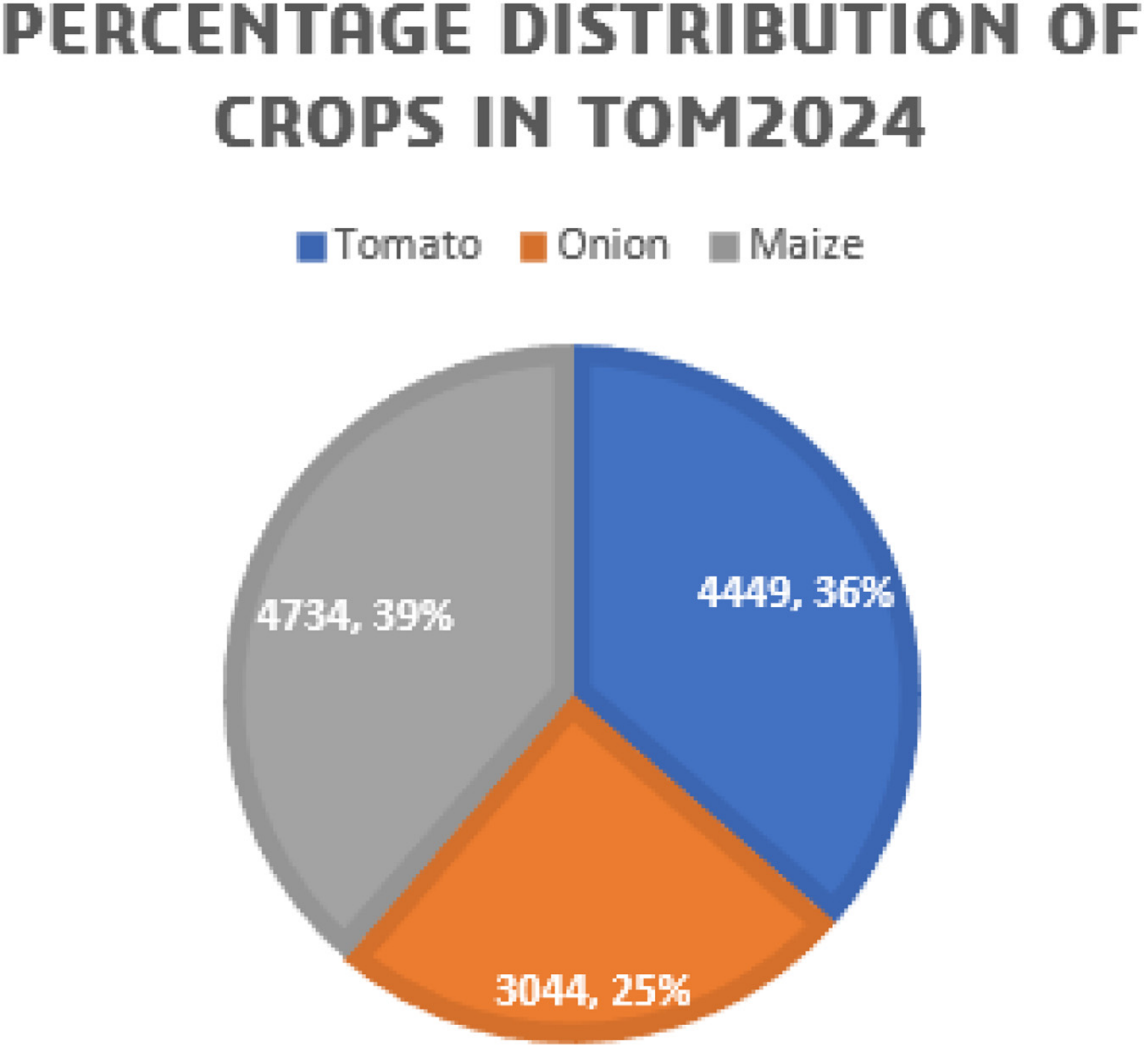
Percentage distribution of crops TOM2024.

**Fig. 3:**
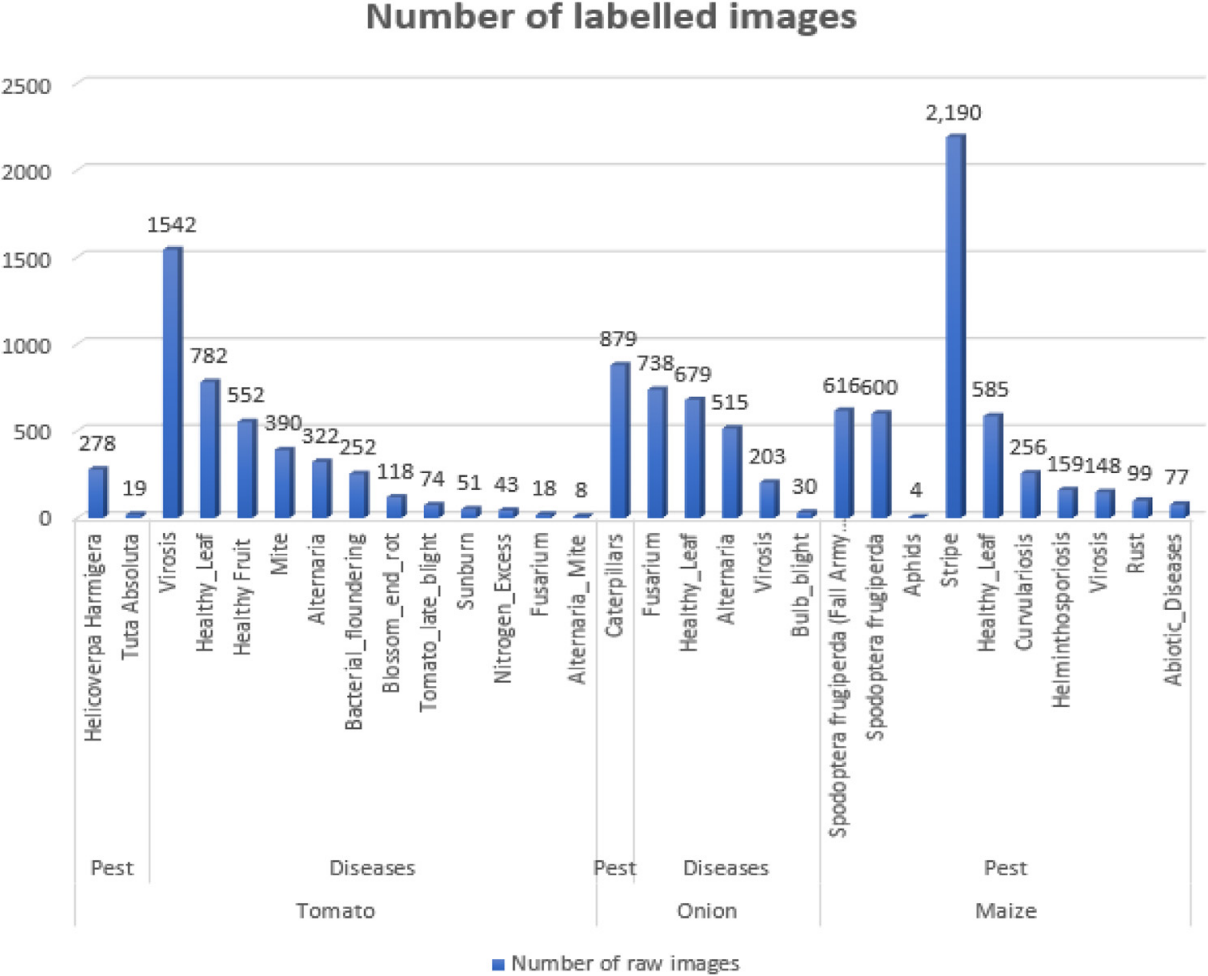
Distribution of labelled images.

**Fig. 4:**
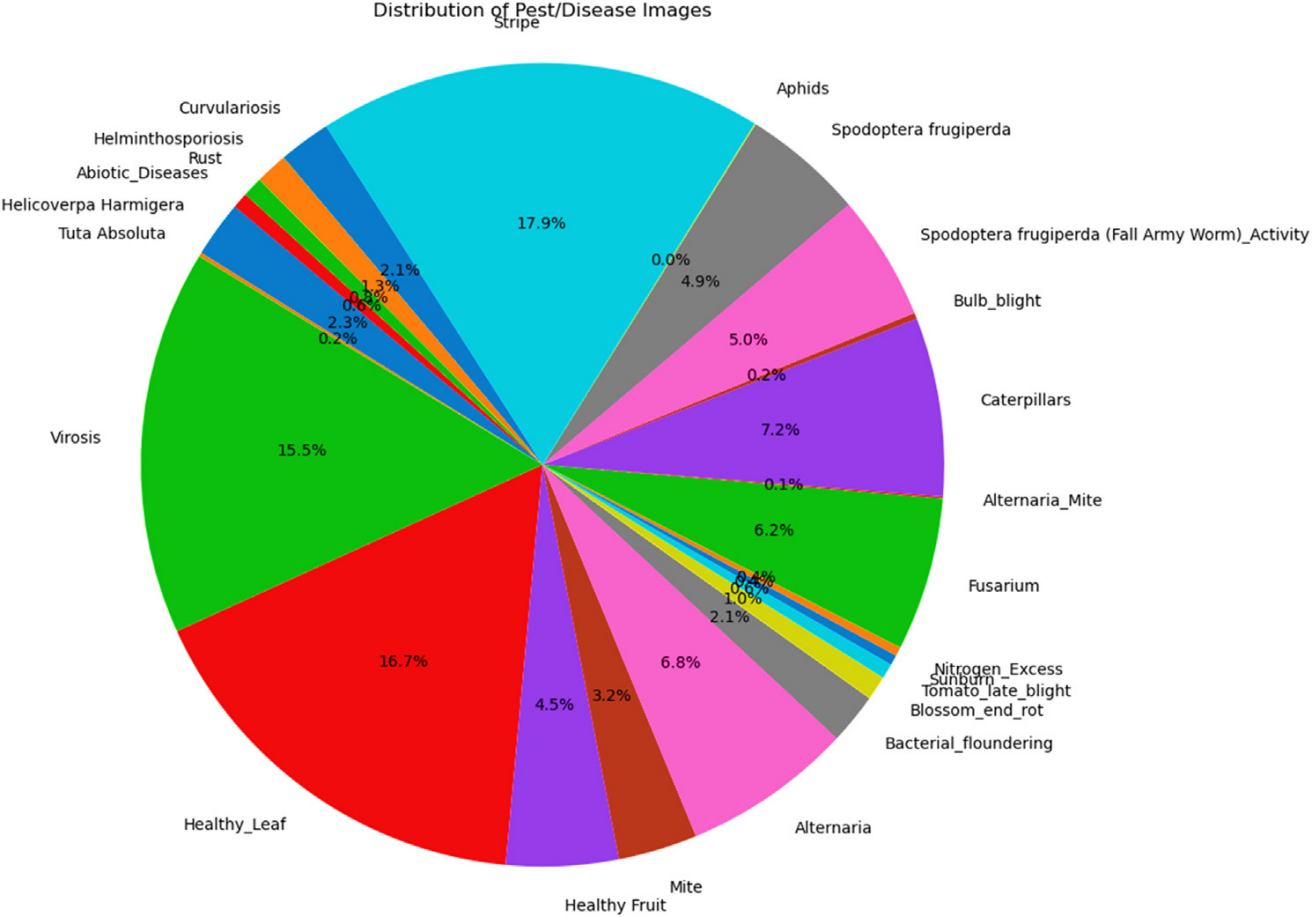
Percentage distribution of all classes in the dataset.

Fig. 2, Fig. 3, Fig. 4 present the total distribution of crops, distribution of classes under each crop and distribution of the various classes captured under the TOM2024 dataset.

#### 3.2.2 TOM2024: category B

**Fig. 5:**
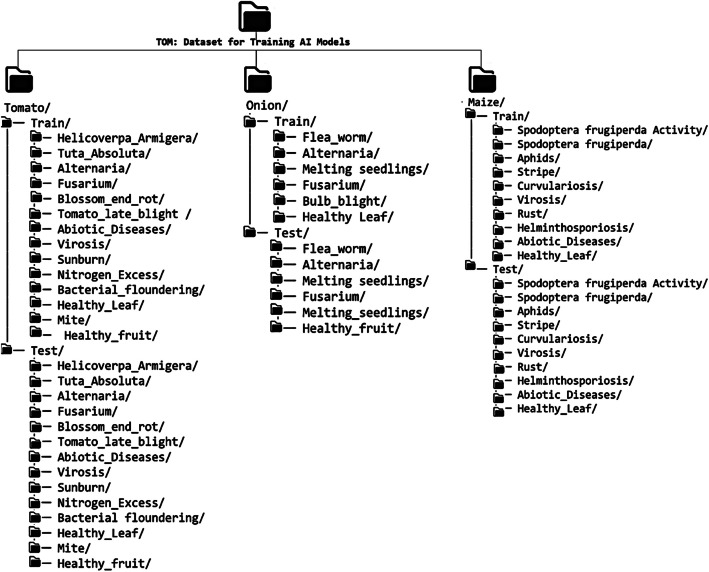
Category B – Sample Dataset for developing AI models.

**Fig. 6:**
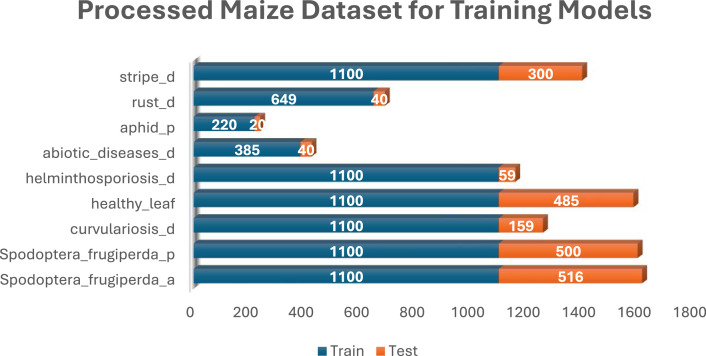
Processed maize dataset for training models.

**Fig. 7:**
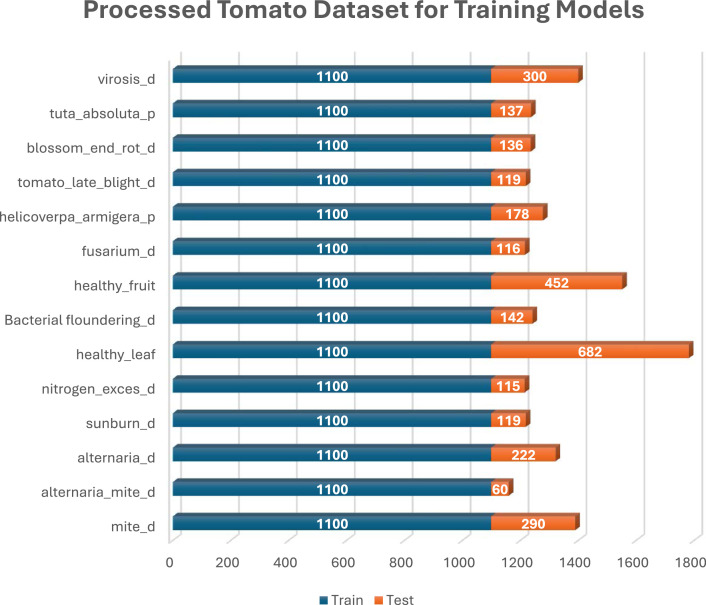
Processed tomato dataset for training models.

**Fig. 8:**
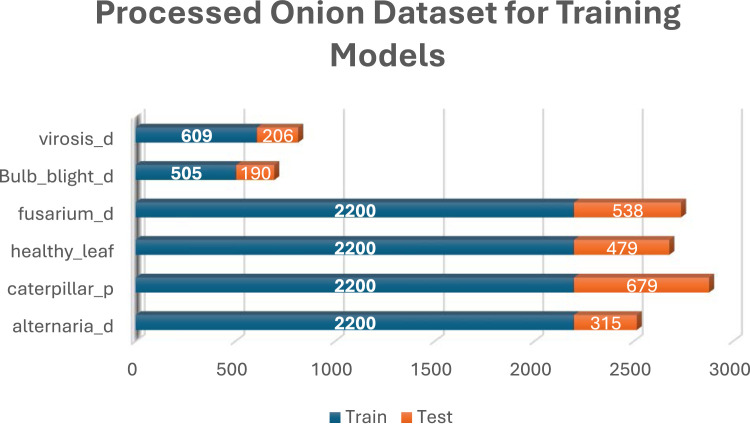
Processed onion dataset for training models.

Category B was created by randomly selecting 100 images from classes with more than 100 images, followed by applying 10 different image augmentation techniques to each selected image. To enhance the diversity and robustness of the dataset, a series of image augmentation techniques were applied using specific parameters. The images were subjected to a rotation range of 15° to introduce variability in orientation, while both width and height shifts were applied within a 0.1 range to simulate positional adjustments. Brightness was varied within a range of [0.5, 1.5] to account for different lighting conditions, and both horizontal and vertical flips were enabled to mirror the images. A zoom range of 0.2 was applied to mimic variations in scale, and a shear range of 0 was used to maintain the image structure. Additionally, images were converted to grayscale using the formula Grayscale = 0.299R + 0.587G + 0.114B to reduce color dependencies. A mean filter with a window size of 7 × 7 was applied to smooth the images, reducing noise and enhancing overall quality. These augmentation techniques were crucial in creating a more robust dataset, capable of supporting advanced machine learning models. Fig. 5 illustrates the data structure of Category B. Fig. 6, Fig. 7, Fig. 8 present the various distribution of images in each of the 3 crops under the Category B dataset.

### 3.3 Category C

**Fig. 9:**
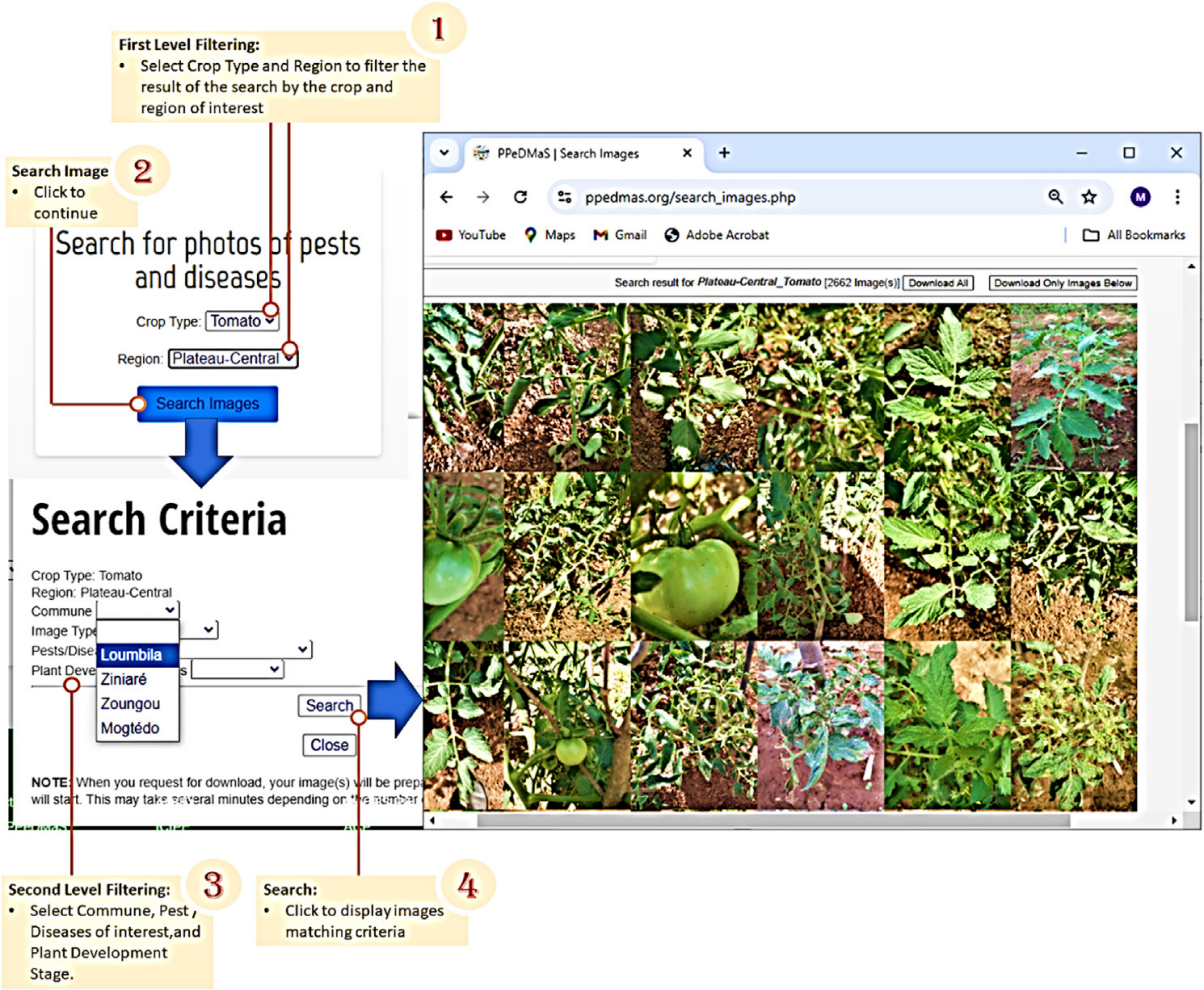
Steps to retrieve images from TOM2024 Category C.

Category C is an online repository that offers the user the opportunity to search through over 25,000 raw images using regions, communes, etc., after which the retrieved images can be downloaded. The datasets are organized into a well-defined hierarchical structure, facilitating ease of access and usability for various applications. Fig. 9 illustrates an interaction with the online TOM2024 Category C

## 4 Experimental Design, Materials and Methods

**Fig. 10:**
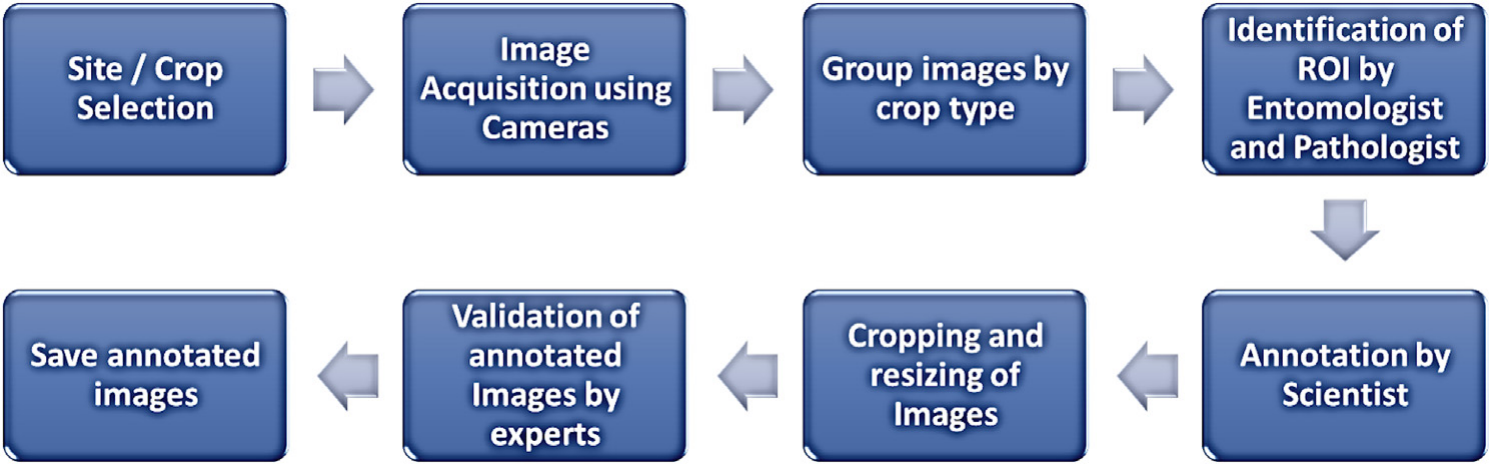
TOM dataset curation process.

The dataset was compiled through a systematic and structured data collection process to capture high-quality images of plant pests and diseases across key crops: maize, tomato, and onion. Fig. 10 illustrates the general procedure employed for the creation of the TOM2024 dataset.

The steps employed for the curation involved the site selection, image acquisition, grouping of images into crop types and issue types (pest or disease), identification of issues by experts, cropping and resizing of images, validation of cropped images by experts, and saving of images online. The section below presents detailed steps employed.

### 4.1 Site selection

**Fig. 11:**
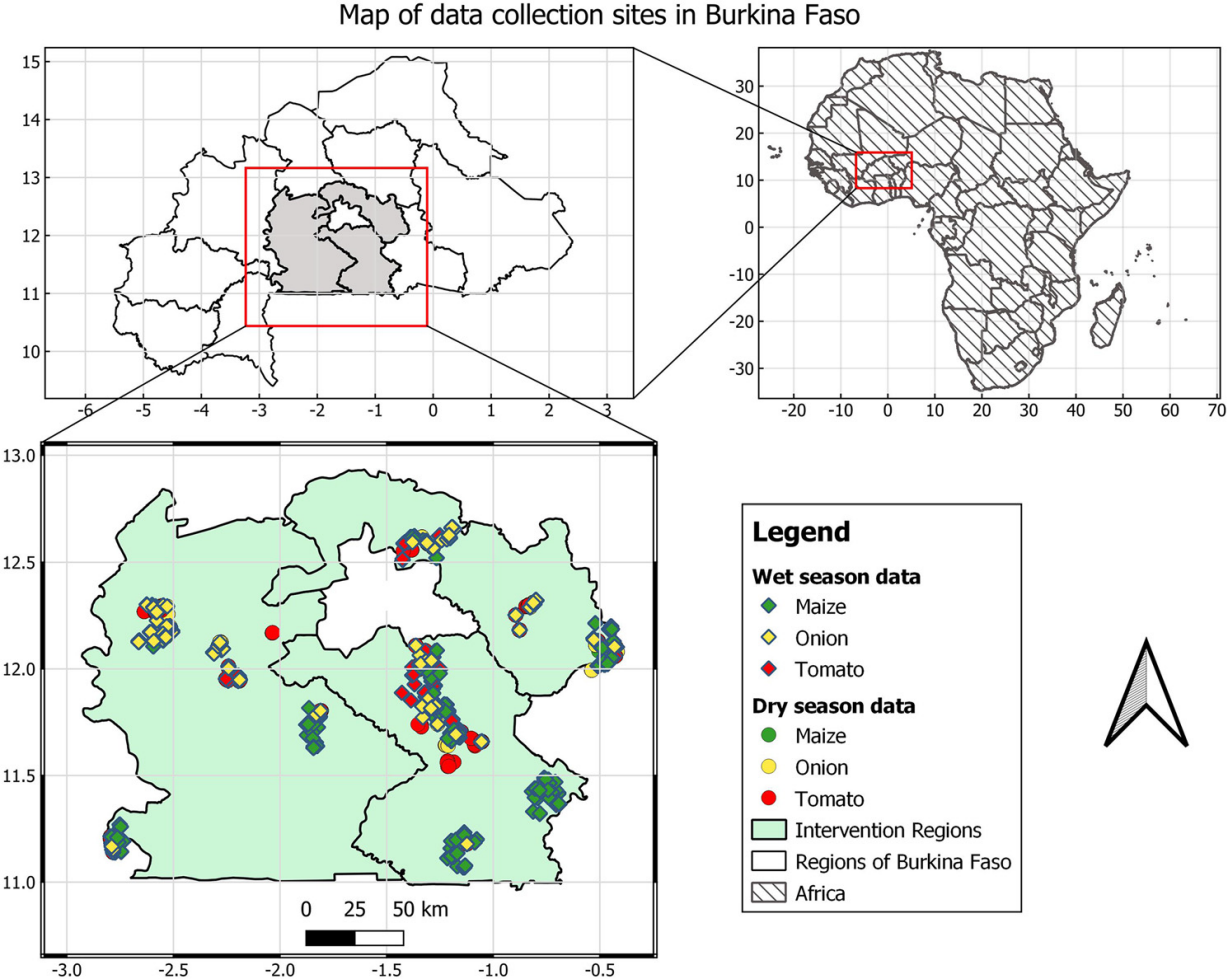
Maps of the exact locations in which images were collected in rainy and dry seasons.

*Geographical Coverage* – The data acquisition process was conducted in Plateau-Central, Centre-Ouest, and Centre-Sud regions in Burkina Faso. This approach was adopted to capture the variability in environmental conditions and crop health across different climatic phases, providing a more comprehensive dataset for analysis and modelling. This multi-region approach was chosen to ensure the diversity and representativeness of the dataset, encompassing distinct geographic and environmental conditions. The selection of farms within these regions was conducted meticulously to ensure an even distribution of data points across different areas. The acquisition of images from farms involved obtaining permission from local farmers. Ethical considerations and proper consent procedures were followed to ensure the cooperation of farmers in capturing images of crop segments impacted by pests and diseases. These permissions were critical for data collection and promoted a collaborative approach with local agricultural communities. Maps in Fig. 11 illustrates the exact locations in which images were collected. *Crops of Interest –* The primary focus was on maize, tomato, and onion—staple crops with significant agricultural importance in the regions studied. These crops were chosen due to their susceptibility to a variety of pests and diseases, making them ideal candidates for building a comprehensive and robust dataset.

### 4.2 Data collection period

The data collection spanned both the dry and wet seasons, from July 2022 to July 2023. This period was selected to capture the variability in pest and disease occurrence throughout the year. By covering different stages of crop development and varying environmental conditions, the dataset reflects a comprehensive range of agricultural challenges faced by farmers.

### 4.3 Image capture

*Equipment Used* – High-resolution digital cameras were utilized to capture images of crop leaves, stems, and fruits exhibiting symptoms of pest infestations or disease. The use of standardized equipment ensured consistency in image quality across all data points. *Data Collectors -* Trained extension officers were responsible for data collection on the selected farms. Equipped with the necessary tools and expertise, these officers accurately identified and documented visible signs of pests and diseases. *Image Protocol –* A standardized protocol was followed for image capture, which involved taking multiple images of the affected areas from different angles and under varying light conditions. This approach ensured that the symptoms were accurately represented, facilitating more effective image analysis.

The images were acquired using many Samsung Galaxy M11 (SM-M115F/DS) phones equipped with specific technical specifications to ensure high-resolution image capture and data accuracy. This phone was selected for its suitability for field-based data collection in agricultural environments. Table 1 provides an overview of the key specifications of the main camera of the Samsung Galaxy M11 (SM-M115F/DS) used in this data acquisition process (Table 2).

**Table 2:** Mobile Phone main camera specifications for image acquisition.

Phone	Samsung Galaxy M11 (SM-M115F/DS)
Back camera (main camera)	Triple camera: 13 megapixels 5MP ultrawide (f/2.2, 14 mm, 115°) 2MP depth (f/2.4)
Main camera resolution	4128 × 3096 pixels
Video recording (primary)	Full HD (1920 × 1080) 30 fps
Flash	LED flash
Focal aperture	f/1.8 (aperture)
Focal length	27 mm lens
Sensor size	1/3.1″ inches
Pixel size	1.12 µm pixel
Autofocus	PDAF: phase detection autofocus
Touch focus	Supported
Image stabilization	EIS: Digital stabilization
Zoom	Only digital zoom
Face/smile detection	Face detection, Smile detection
HDR	HDR photo on both cameras

The mobile phones were employed by a team of extension officers who conducted image acquisition across various farms in the three regions of Burkina Faso. To ensure the highest quality of data, the process of capturing images of crop pests and diseases followed a structured approach. Extension officers meticulously captured images of plant segments impacted by diseases or pests. The process included the selection of various crop development stages and the careful examination of crops under different lighting conditions and backgrounds, such as white, dark, illuminated, and natural settings. This systematic approach aimed to minimize any variations in image quality, providing a comprehensive dataset for accurate pest and disease identification and facilitating the development of effective digital solutions for crop management.

### 4.4 Annotation and labeling

*Manual Annotation –* The images collected were manually annotated by agricultural experts to identify and label the specific pests and diseases present. This process involved cross-referencing with established agricultural guidelines and visual symptom libraries to ensure accuracy. *Label Categories –* The dataset was categorized into distinct classes based on the type of pest or disease. Additionally, healthy plant images were included for comparison. Each image was labelled with metadata, including the crop type, the part of the plant affected, and the specific pest or disease identified.

### 4.5 Quality control

*Verification* – To ensure annotation accuracy, a subset of images was reviewed by a second team of experts. Any discrepancies were resolved through consensus, and images with uncertain or ambiguous labels were excluded from the final dataset. *Consistency Checks* – Regular checks were conducted throughout the data collection process to maintain consistency in image quality and labeling accuracy. Images that did not meet the quality standards were either retaken or discarded.

### 4.6 Data storage and organization

*File Structure* – The dataset was organized into a hierarchical directory structure, with folders representing each crop and subfolders for different pests and diseases. This organization facilitated easy access and retrieval of specific data subsets. *Metadata* – Each image was accompanied by a metadata file containing relevant information such as the date of capture, geographic location, crop type, and label details. This metadata is crucial for contextualizing the images and supporting subsequent analysis.

### 4.7 Potential use cases and applications

The dataset developed and utilized in this study holds significant potential for a wide range of applications in both research and industry, particularly within the realms of artificial intelligence (AI) and machine learning (ML). By leveraging the rich and diverse data available, AI/ML models can be trained to accurately identify and classify various pests and diseases affecting key crops such as onions, tomatoes, and maize. The dataset's comprehensive coverage across different regions and seasons further enhances the robustness of these models, making them applicable to a broad spectrum of agricultural contexts.

TOM2024 – Category B, in particular, was instrumental in developing the Proposed Framework [15], which achieved a remarkable accuracy of over 96 %. In the paper *PlanteSaine: An Artificial Intelligent Empowered Mobile Application for Pests and Disease Management for Maize, Tomato, and Onion Farmers in Burkina Faso* [15], the relevancy of the created dataset is demonstrated. In addition to it models development applications, this dataset can serve as a valuable resource for academic research and the advancement of precision agriculture technologies. Its use in industrial settings could lead to the development of automated monitoring systems, integrated pest management tools, and other innovations that enhance the sustainability and productivity of farming practices.

## Limitations

The TOM2024 dataset, while comprehensive, has limitations that may impact its effectiveness in diverse applications. First, it is geographically specific, meaning the data primarily reflects crops, pests, and diseases from certain regions, which may not generalize well to other areas with different environmental conditions. Additionally, the diversity of crop varieties in the dataset is limited, potentially affecting the performance of AI models when applied to less common varieties. Another issue is the variation in image quality, with differences in lighting, resolution, and focus that can introduce noise and hinder model accuracy. The dataset also tends to focus on common pests and diseases, leading to an imbalance that may reduce the ability of AI models to identify rare or emerging threats. Moreover, the dataset lacks detailed contextual information, such as crop growth stages or environmental factors, which are crucial for accurate diagnosis. While data augmentation expands the dataset, it may introduce artifacts that don't reflect real-world conditions, limiting the generalizability of models. Finally, the dataset is static and may not adapt to evolving pest and disease dynamics, further restricting its long-term applicability.

## Ethics Statement

The dataset used in this study was collected from farms where the farmers provided their consent for participation. A consent form was presented to the farmers, and upon their approval, they signed the form, granting permission for data collection on their farms

## CRediT Author Statement

**Obed Appiah:** Data curation, Conceptualization, Supervision, Original Draft; **Kwame Oppong Hackman:** Data curation, Conceptualization, Supervision, Original Draft; **Son Diakalia:** Validation, Writing - reviewing and editing; **Audrey Kantz Dossou Codjia:** Methodology, Software, Writing –original draft; **Momo Bêbê:** Data curation, Validation; **Valentin Ouedraogo:** Methodology, Software, Writing –original draft; **Belko Abdoul Aziz Diallo:** Data curation, Conceptualization, Supervision, Original Draft; **Kisito Gandji:** Writing - reviewing and editing; **Damoue Abdoul-Karim:** Writing - reviewing and editing; Kehinde Olufunso **Ogunjobi:** Data curation, Validation, Supervision; **Gaston Dabire:** Writing - reviewing and editing; **Charles Lamoussa Sanou:** Validation, Writing - reviewing and editing; **David Anaafo:** Writing - reviewing and editing; **Emmanuel Ramde:**Validation and Supervision*.*

## Data Availability

Mendeley DataTOM2024 (Original data).
